# Analysis of the Differentially Expressed Proteins and Metabolic Pathways of Honeybush (*Cyclopia subternata*) in Response to Water Deficit Stress

**DOI:** 10.3390/plants12112181

**Published:** 2023-05-31

**Authors:** Mary-Jane S. Mahlare, Lizex Husselmann, Muinat N. Lewu, Cecilia Bester, Francis B. Lewu, Oluwafemi James Caleb

**Affiliations:** 1 Agricultural Research Council (ARC) Infruitec-Nietvoorbij, Stellenbosch 7599, South Africa; 2 Department of Agriculture, Faculty of Applied Sciences, Cape Peninsula University of Technology, Wellington Campus, Private Bag X8, Wellington 7654, South Africa; 3 Department of Biotechnology, University of the Western Cape, Bellville 7535, South Africa; 4 Africa Institute for Postharvest Technology, Faculty of AgriSciences, Stellenbosch University, Private Bag X1, Matieland 7602, South Africa; 5 Department of Horticultural Science, Faculty of AgriSciences, Stellenbosch University, Matieland 7602, South Africa

**Keywords:** *Cyclopia subternata*, differentially expressed proteins (DEPs), water deficit stress, carbon fixation, proteomic analysis

## Abstract

Honeybush (*Cyclopia* spp.) is a rich source of antioxidant properties and phenolic compounds. Water availability plays a crucial role in plant metabolic processes, and it contributes to overall quality. Thus, this study aimed to investigate changes in molecular functions, cellular components, and biological processes of *Cyclopia subternata* exposed to different water stress conditions, which include well-watered (as Control, T1), semi-water stressed (T2), and water-deprived (T3) potted plants. Samples were also collected from a well-watered commercial farm first cultivated in 2013 (T13) and then cultivated in 2017 (T17) and 2019 (T19). Differentially expressed proteins extracted from *C. subternata* leaves were identified using LC-MS/MS spectrometry. A total of 11 differentially expressed proteins (DEPs) were identified using Fisher’s exact test (*p* < 0.00100). Only α-glucan phosphorylase was found to be statistically common between T17 and T19 (*p* < 0.00100). Notably, α-glucan phosphorylase was upregulated in the older vegetation (T17) and downregulated in T19 by 1.41-fold. This result suggests that α-glucan phosphorylase was needed in T17 to support the metabolic pathway. In T19, five DEPs were upregulated, while the other six were downregulated. Based on gene ontology, the DEPs in the stressed plant were associated with cellular and metabolic processes, response to stimulus, binding, catalytic activity, and cellular anatomical entity. Differentially expressed proteins were clustered based on the Kyoto Encyclopedia of Genes and Genomes (KEGG), and sequences were linked to metabolic pathways via enzyme code and KEGG ortholog. Most proteins were involved in photosynthesis, phenylpropanoid biosynthesis, thiamine, and purine metabolism. This study revealed the presence of trans-cinnamate 4-monooxygenase, an intermediate for the biosynthesis of a large number of substances, such as phenylpropanoids and flavonoids.

## 1. Introduction

Honeybush is the general term used to classify all known types of *Cyclopia* species, a genus in the leguminous family [1]. It is native to the fynbos biome in the Western and Eastern Cape Provinces of South Africa, and its tea has a sweet, honey-like taste [2]. *Cyclopia* species are among the few wild plants that have been turned into commercial commodities in South Africa. Although 23 species grow in the wild, only a few are currently used to make tea, including *C. subternata*, *C. intermedia*, *C. genistoides*, *C. maculata*, *C. longifolia,* and *C. sessiliflora* [3,4]. The popularity of honeybush can also be attributed to its low tannin content, the absence of caffeine, and the presence of antioxidants. The tea from honeybush is usually enjoyed in its fermented state, although the unfermented (green) one is also marketed [5]. These herbal teas are famous for their rich organic antioxidant properties and phenolic compounds, which are helpful in treating colon, throat, and lung ailments, heartburn, ulcers, nausea, and urinary tract infections [6,7]. Polyphenols in the unfermented honeybush tea have been broadly perceived as having anti-cancerous properties [8,9].

Increasing awareness of the health benefits of *Cyclopia* species is one of the factors contributing to the market growth of the tea. Extracts of honeybush teas are also utilized in food and other aesthetic products [10]. During the last two decades, the export of South African honeybush has expanded due to an increase in demand. It is estimated that about 90% of the honeybush tea crop is exported, with the largest share to the Netherlands (±44%), Germany (±30%), United Kingdom (±8%), and the United States (±7%), while the remaining percentage is packaged for local consumption in South Africa [11,12].

Plants are continually exposed to biotic and abiotic stresses in their natural and agricultural environments, which can threaten their survival and growth [13]. Stressful conditions could result in delayed seed germination, reduced plant growth, and lower crop yield [14,15]. Furthermore, a water deficit could inhibit essential processes such as photosynthesis, respiration, and transpiration, thus restraining the growth and development of plants [16,17]. Lack of water can alter plant chemistry and negatively influence their growth by disrupting metabolic processes and pigment formation, thereby decreasing photosynthesis, and thus preventing food production [18,19,20]. Heat and water stress have the potential to compromise sustainable crop production on a global scale [21,22]. Plants can be affected by water stress in two different ways, drought (too little water) or waterlogging (too much water), which reduce oxygen in the soil and impair nutrient uptake [23,24].

There are numerous molecular programs in plants such as drought avoidance, drought tolerance, drought escape, proline accumulation, synthesis of proteomes, accumulation of osmolytes, and closure of stomatal conductance, which have evolved in response to these changes in available water. These molecular programs quickly detect and adapt to environmental changes and other stresses [3,25,26]. Plant proteins play a crucial role in both biotic and abiotic stress responses because they regulate physiological characteristics directly to adapt to changes in the environment. They are also crucial executors of cellular mechanisms and key players in cellular homeostasis [27]. The scarcity of genomic information has hampered crop proteomics applications. However, within the past decade and due to the advancement in analytical tools, the use of proteomics tools for crop plant analysis has amplified drastically. With the successful development of “next generation” sequencing technology, the identification and annotation of proteins and their isoforms in a specific crop species are becoming considerably easier [14].

Proteomics analysis aims to provide an inclusive profile of various proteins found in a specified organism in response to different biotic or abiotic stresses [3,28,29]. Plant resistance mechanisms [27,30,31], responses to water stress [32,33,34], and other exogenous stimuli [35] have been investigated using proteomics tools in several studies. For example, Haddoudi et al. [34] investigated the morpho-physiological, biochemical, and molecular responses to water deficit stress in four contrasting *Medicago truncatula* lines (TN6.18, JA17, TN1.11, and A10). The study showed that line TN6.18 was most resistant to water deficit stress with the highest root biomass production, a significantly higher increase in soluble sugar and its total protein contents, and lower levels of lipid peroxidation with greater cell membrane integrity. Furthermore, RT-qPCR revealed that the DREB1B gene had a higher induction rate in roots of TN6.18 and JA17 than in A10 roots. The authors suggested that DREB1B plays a key role in the water deficit tolerance of *M. truncatula*. However, there is a limited understanding of how potted and commercially farmed *C. subternata* plants would respond to induced/natural water stress at the proteomic level. Therefore, the aim of the present study was to compare the effect of water deficit stress on molecular functions, cellular components, and biological processes of potted and commercially farmed *C. subternata* plants. This study provides insights into the proteomic basis of drought tolerance mechanisms mediated by the key regulators.

## 2. Results

### 2.1. 1D-SDS-PAGE of C. subternata Protein Samples

**Figure 1:**
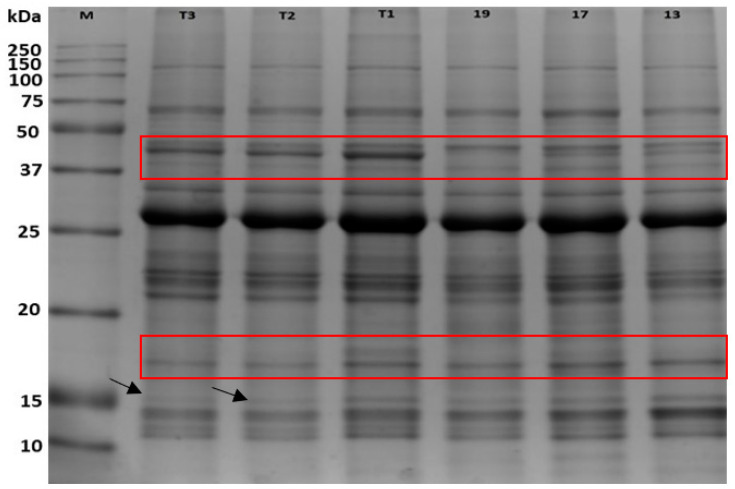
A representative one-dimensional electrophoresis gel profile of Cyclopia subternata leaf protein in response to different water stress treatments. Approximately 20 µg of protein sample were loaded on a 12% SDS-PAGE electrophoresis gel. M = Molecular weight marker (ladder); T1 = Nietvoorbij well-watered plant sample; T2 = Nietvoorbij semi-stressed plant sample; T3 = Nietvoorbij water-deprived plant sample; and T-19, -17, and -13 = Napier well-watered plants cultivated in 2019, 2017, and 2013, respectively. The red box and black annotated arrows indicate marked changes in protein intensity.

One-dimensional gel electrophoresis showing the responses of *C. subternata* protein samples to different water stress levels is presented in Figure 1. Protein loading was relatively uniform, and no streaking of proteins was observed in the leaf protein extracts. The information integrates the molecular weight and the amount of protein extract per treatment. Protein bands from all treatments covered a molecular weight range of 10 to 150 kDa. The youngest commercial farm samples (T19) have a missing band between 37 and 50 kDa. In contrast, an extra protein that differentiates T1 and T17 from the other treatments was located between 15 and 20 kDa. Similarly, at 15 kDa, the two water-stressed potted plants had lower band intensity (Figure 1).

### 2.2. Identification of Induced Proteins in C. subternata Using LC-MS/MS Analysis

Based on known proteins, over 600 proteins were identified across all the treatments; however, after the four filter standards (Fisher’s exact test at *p* < 0.00100, peptide threshold (95%), protein threshold (1% false discovery rate (FDR), and a minimum of two peptides) used, the results showed that only 11 differentially expressed proteins (DEPs) were found to be significant (*p* < 0.00100). Table 1 presents significantly different proteins identified from stressed and well-watered treatments in *C. subternata* leaves. For example, a total number of 269 proteins were identified from the broad comparison of both well-watered *C. subternata* plants (T17 and T19) that were cultivated commercially. However, only one enzyme (α-glucan phosphorylase) was found to be statistically common between T17 and T19 (*p* < 0.00100) using Fisher’s exact test. Notably, the enzyme (α-glucan phosphorylase) was upregulated in T17 (older vegetation) and downregulated in T19 by 1.41-fold. This result suggests that α-glucan phosphorylase was produced less in T19 and more in T17 as needed.

### 2.3. GO Analysis of Gene Ontology Enrichment

**Figure 2:**
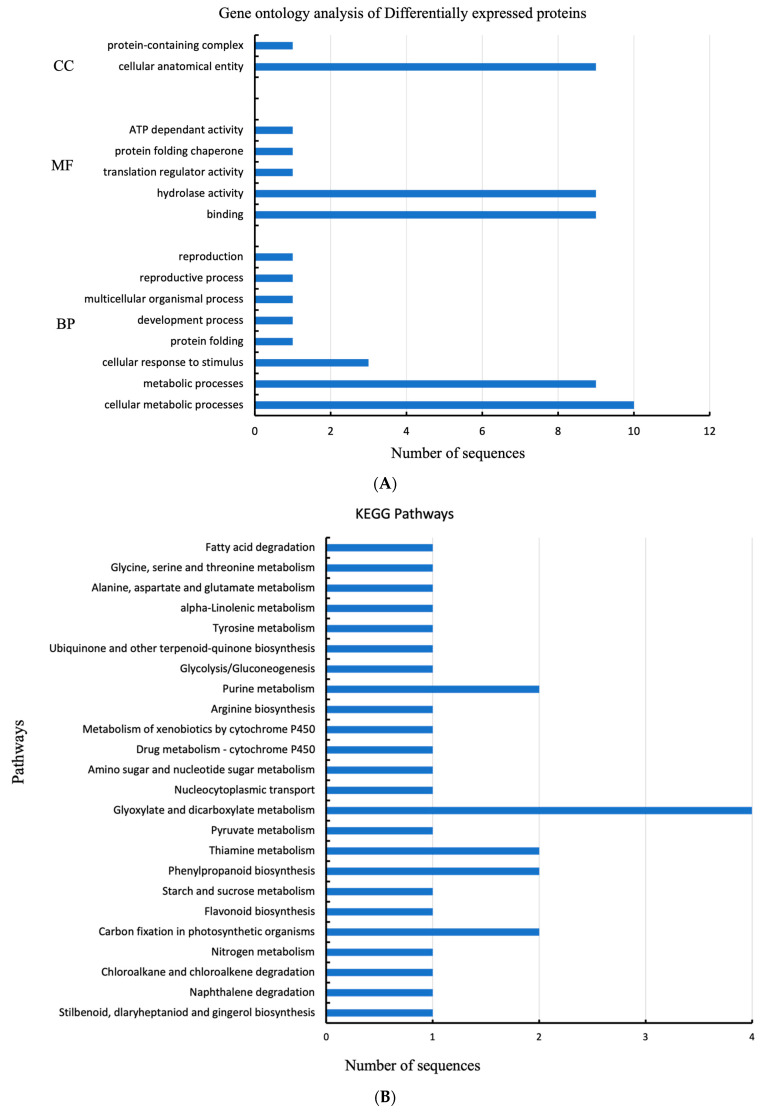
(**A**) Gene ontology (GO) analysis of common (shared) proteins differentially regulated in Cyclopia subternata leaf under water deficit stress and regularly watered as determined by Panther (http://www.pantherdb.org, accessed on 22 August 2022) (according to GO distribution by level 1. BP = biological processes; MF = molecular functions; CC = cellular components. (**B**) An illustration of metabolic pathways linked to differentially expressed proteins in the leaves of *C. subternata* under water deficit stress as well as regularly watered as retrieved by Kyoto Encyclopedia of Genes and Genomes (KEGG) pathway analysis via EggNog-Mapper.

Differentially expressed proteins involved in combined water deficit with heat stress were mapped using Panther for gene ontology analysis (http://www.pantherdb.org, accessed on 22 August 2022). Based on the total number of proteins listed above in Table 1, the Panther analysis annotated into three categories of GO distribution by level 1, namely, biological process (BP), molecular function (MF), and cellular component (CC). The proteins involved in these activities include α-glucan phosphorylase, ribulose bisphosphate carboxylase, trans-cinnamate 4-monooxygenase, UDP-arabinopyranose mutase 1, cinnamyl alcohol dehydrogenase, glutamine synthetase nodule isozyme, elongation factor 1-α, and chlorophyll a-b binding protein. This was to improve the understanding of biological functions associated with water stress in C. subternata. Figure 2A summarizes the gene ontology distribution of proteins obtained from treated (water-stressed) and non-treated (well-watered) C. subternata leaf samples.

### 2.4. Kyoto Encyclopedia of Genes and Genomes (KEGG) Pathway Analysis

Proteins were mapped and assigned to different metabolic pathways according to the KEGG pathway database for a better understanding of the roles of DEPs in *C. subternata* leaves (Figure 2B). Sequences were linked to pathways via both enzyme code (EC) and KEGG ortholog (KO). According to the results of this study, a total of 30 sequences were associated with 24 KEGG pathways, where only five were differentially expressed. The KEGG pathway database was used to sort the identified metabolic pathways into major categories.

## 3. Discussion

### 3.1. 1D-SDS-PAGE and LC-MS/MS Analysis

Based on protein fragmentation via SDS-PAGE, similar banding patterns or fragmentation with varying abundance or intensity were observed across the different water treatments for *C. subternata*. However, as indicated by the intensity of the bands, the treated samples from the Nietvoorbij farm glasshouse (T1–T3) had greater protein abundance between 37 and 50 kDa compared with all other treatments (Figure 1). In contrast, band intensity at the lower molecular weight range between 10 and 15 kDa was more expressed in commercial farm samples (T13 to T19) than in other treatments. However, the youngest commercial farm samples (T19) have a missing band between 37 to 50 kDa, while T1 had the highest intensity in one of the bands within this range. In contrast, an extra protein that differentiates T1 and T17 from the other treatments was located between 15 and 20 kDa, but the intensity of this band also differed between the treatments. Similarly, at 15 kDa the two water-stressed potted plants had lower band intensity (Figure 1). The differences in fragmented protein abundance suggest that there were possible events of either up- or downregulation of various proteins within the molecular weight range [36].

According to Ubiparip et al. [37], α-glucan phosphorylases play an essential role in catalyzing reversible phosphorolysis and storing polysaccharides such as glycogen, starch, and maltodextrins. This could suggest that T17, which is the older plant, is well adapted for the storing of available/excess polysaccharides compared to the younger plants. The reason for the presence of α-glucan phosphorylase in these samples alone may be because they were irrigated at equal rates. Furthermore, the comparison of the total number of proteins identified between well-watered T1, cultivated in the glasshouse, and that well-watered and commercially farmed (T19) was 271. However, only four proteins (i.e., ribulose bisphosphate carboxylase large chain, (RubisCO), trans-cinnamate 4-monooxygenase, probable UDP-arabinopyranose mutase 1, and cinnamyl alcohol dehydrogenase) were found to be significantly common between both samples (*p* < 0.00100). The cluster of one RubisCO large subunit protein was found to be 2.41-fold higher in T1 than in T19. RubisCO is the key enzyme that facilitates the process of carbon fixation in the Calvin cycle. The fold increase under T1 (well-irrigated) could suggest that there was a higher photosynthetic carbon flux in the Calvin cycle under the glasshouse conditions. The results found in this study contradict those in the study by Haworth et al. [38] on olives, where heat stress stimulated a deterioration in photosynthesis with reduced RubisCO activity.

Furthermore, the downregulation of RubisCO subunits under T19 could suggest nitrogen deficiency in the leaf [39]. Abiotic stresses such as drought reduce the rate of photosynthesis by disturbing the cell homeostasis and affecting the photosynthetic pigments, soluble proteins, proteins in thylakoid membranes, the electron transport chain, photophosphorylation, and carbon dioxide (CO_2_) fixation [40]. During drought conditions, crop growth and yields are seriously impaired, and photosynthesis is hindered [41]. Based on the results obtained from T1 and T19 protein profiles, the impact of abiotic stress due to water deficit/stress could not be established. Probable cinnamyl alcohol dehydrogenase (CAD) was downregulated in T1 and upregulated in T19 by 1.06-fold. According to reports, cinnamyl alcohol dehydrogenase catalyzes the last stages of the production of monolignol [42]. Lignins are complex polymers that play a role in the alteration of biofuel and ensure good leaf quality in plants [43]. They also play an essential role in plant defense, mechanical support, and water retention [44]. Different plants have responded differently to CAD downregulation in terms of lignin content [43]. Therefore, the downregulation of CAD enzyme in this study may suggest that a low amount was needed in T1 (Nietvoorbij well-watered plants) compared to T19 (Napier commercial farm plants). The fold change in probable UDP-arabinopyranose mutase 1 was reported to be 1.07 times lower in T1 than in T19. Saqid et al. [45] stated that L-Arabinofuranose is an omnipresent component produced from the cytosolic UDP-arabinopyranose (UDP-Arap mutase 1). In contrast, trans-cinnamate 4-monooxygenase had a fold change of 1, with T1 being lower and T19 being higher. Trans-cinnamate is a naturally occurring aromatic compound in plants, and it could serve as a central intermediate for the biosynthesis of a large number of substances, such as phenylpropanoids, coumarins, and flavonoids [46]. Enzymes such as trans-cinnamate 4-monooxygenase (CYP73A) were identified in the ‘phenylpropanoid biosynthesis’ pathway in [47].

Based on known proteins and the four filter standards used, the results showed that only six differentially expressed proteins (DEPs) were found between the T19 (well-irrigated treatment) and T3 (stressed treatment from Nietvoorbij) groups. This included the four enzymes (three large chain ribulose bisphosphate carboxylase subunits and glutamine synthetase (GS) nodule isozyme) and two proteins (chlorophyll a/b binding protein, chloroplastic and elongation factor 1-alpha). Glutamine synthetase plays an important role in the metabolism of nitrogen by catalyzing the reaction of condensation of glutamate and ammonia to form glutamine [48]. The GS enzyme was downregulated in the stressed treatment (T3) by 1.62-fold compared to the well-watered treatment (T19) in response to water stress in this study. The findings are comparable to those reported by Mabizela [3] on *C. subternata*. During drought stress, nodule function and the growth of honeybush are directly affected when plants receive less water during summer periods, as honeybush are rain-fed plants. Furthermore, drought decreases the rate of photosynthesis and lowers the level of photosynthates needed by the bacteria for nitrogen fixation [49]. This finding may suggest that there was a positive nitrogen metabolism, which may be true as *Cyclopia* species are known to fix their own nitrogen [50,51].

Chloroplast is responsible for both light and dark reactions during photosynthesis, but it is highly sensitive to various abiotic stresses [52]. Drought disrupts cellular homeostasis and affects the photosynthetic pigments, soluble proteins, proteins in the thylakoids membranes, the electron transport chain, photophosphorylation, and carbon dioxide (CO_2_) fixation, which reduces the rate of photosynthesis. The closure of stomata also decreases photosynthesis, increasing chloroplast and sub-stomatal CO_2_ concentration and decreasing CO_2_ assimilation [40]. There was a downregulation of chlorophyll a/b binding proteins in the stressed treatment (T3) compared to T19. Drought causes leaf stomatal closure, which hinders CO_2_ entry into the mesophyll cells, thus reducing photosynthesis [53]. This is supported by a study conducted by Mabizela [3], where the amount of chlorophyll a/b-binding proteins in plants increased under drought conditions. In agreement, Benešová et al. [54] reported an increase in chlorophyll a/b-binding protein levels in a tolerant genotype of *Zea mays*, leading to open stomata and efficient transpiration. According to the proteomic findings, *C. subternata*’s photosynthesis-related proteins are regulated during drought stress, which may have crucial implications for plant tolerance research.

### 3.2. Gene Ontology Enrichment and KEGG Pathway Analysis

The reported DEPs were mostly found in the biological processes of cellular process, metabolic process, and response to a stimulus. Additionally, the biological processes involved are growth, developmental, multicellular organismal, reproductive, and reproduction processes. In terms of molecular functions, most of the differently expressed proteins identified were involved in binding and catalytic activities in leaves. Some of the observed molecular functions include translation regulator activity, protein folding chaperone, and ATP-dependent activity. In terms of the cellular component category, the most differentially expressed proteins identified were in the cellular anatomical entity followed by the protein-containing complex.

The enriched KEGG pathways in Figure 2B demonstrate that the greatest number of enzymes annotated in the leaves of *C. subternata* were linked to the glyoxylate and dicarboxylate metabolism. This may suggest that the activation of this pathway plays a crucial role in the mechanisms of water stress responses in *C. subternata*. Some of the observed metabolic pathways that were also identified in the leaves are carbon fixation in photosynthetic organisms, phenylpropanoid biosynthesis, thiamine metabolism, and purine metabolism.

### 3.3. Regulation of Biosynthesis of Secondary-Metabolites-Related Pathways

#### 3.3.1. Phenylpropanoids Pathway

**Figure 3:**
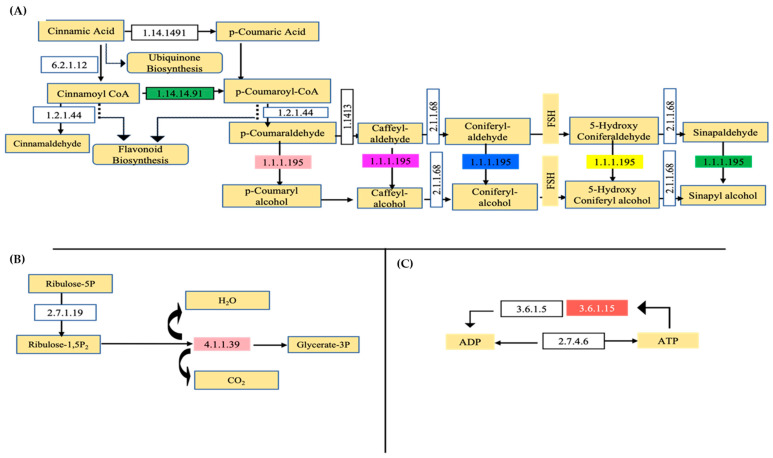
KEGG pathway analysis revealing proteins differentially regulated in water deficit plants of *C. subternata*. (**A**) Differentially expressed proteins identified, i.e., E.C 1.14.14.91 (trans-cinnamate 4-monooxygenase) and E.C 1.1.1.195 (cinnamyl-alcohol dehydrogenase), affecting the phenylpropanoid biosynthesis pathway are shown by colored blocks. (**B**) The enzyme (E.C.4.1.1.39—ribulose-bisphosphate carboxylase) shown by colored block affecting the conversion of ribulose-1,5P_2_ to glycerate-3P in the carbon fixation in photosynthetic organism pathway. (**C**) In purine metabolism pathway, the enzyme identified is shown by red colored block, with relevant enzyme code E.C 3.6.1.15, nucleoside-triphosphate phosphatase in conjunction with E.C. 3.6.1.5 (ATP-diphosphatase) affecting the conversion of ATP to ADP.

Environmental stresses have the tendency to hinder plant growth and productivity by altering the metabolism of reactive oxygen and nitrogen species [55]. Plants under water stress experience oxidative stress, which changes how phenylpropanoids, flavonoids, and other secondary metabolites are synthesized. To detoxify ROS, plant cells evolve an antioxidant enzymatic defense system that uses both enzymatic and non-enzymatic antioxidants [55]. One of the most widely studied metabolic pathways among secondary metabolites is the phenylpropanoids pathway [56]. Metabolites from phenylpropanoid pathways are important for plant development, structural support, and responsiveness to internal and external stimuli. These metabolites are crucial mediators of plants’ interactions with other organisms and play a significant role in stress response to light variations [57] and mineral scarcity [58]. The phenylpropanoids pathway (ko00940) in the leaves of *C. subternata* contains the largest concentration of differentially expressed proteins in secondary metabolism (Figure 3a). From this pathway, two proteins were identified, namely, cinnamyl-alcohol dehydrogenase (EC:1.1.1.195), which is the most dominant, and heme-thiolate (EC:1.14.14.91). These results are comparable to studies by Yu et al. [59], where increased cinnamic acid accumulation and tolerance were imparted to millet through the regulation of genes involved in the phenylpropanoid biosynthesis pathway under drought stress.

#### 3.3.2. Carbon Fixation in Photosynthetic Organism

Nearly all biological processes on earth rely on autotropic CO_2_ fixation, which has created prehistoric carbon reserves that are used today to meet more than 80% of the world’s energy needs [60]. The most biologically prevalent and commercially significant method for carbon fixation is the reductive pentose phosphate pathway (Calvin cycle), which has attracted most of the research attention [61]. Since ancient times, many plant species have been bred to increase their agricultural and commercial value. However, instead of increasing photosynthetic efficiencies, these methods typically produce varieties with a higher percentage of biomass directed towards a given product such as edible seeds or fruits [60].

During the onset of drought, a reduction in stomatal conductance can reduce the availability of CO_2_ for photosynthesis, subsequently leading to the inhibition of underlying biochemical processes such as Rubisco carboxylation and electron transport activity, relative water content, and even pigment content [62]. In this study, analyses of proteins related to the carbon fixation in photosynthetic organism pathway (ko00710) showed that the enzyme ribulose biosphosphate caroboxylase (EC:4.1.1.39) was differentially expressed in the leaves of *C. subternata* that were cultivated under different water stress conditions (Figure 3b). The most prevalent enzyme in the biosphere, rubisco, is one of the commonly known enzymes; it is the main carboxylase of the photosynthetic process. Carboxylation of ribulose bisphosphate is the initial step in the photosynthetic carbon reduction cycle, which results in the uptake of CO_2_ [63].

#### 3.3.3. Purine Metabolism Pathways

Purine and pyridine serve as building blocks to produce nucleic acids and as an energy source. Therefore, nucleic acid biosynthesis and metabolism play a key role in the growth and development of plants [64]. The salvage process is used to use preformed purine bases and nucleosides for nucleotide synthesis. In some cases, purine nucleosides come from exogenous sources as catabolic products of nucleic acids in decomposing cells. Purine nucleosides result from the intercellular breakdown of unstable RNA [65,66]. In this study, analyses of proteins related to the purine metabolism pathway (ko00230) showed that an enzyme nucleoside-triphosphate phosphatase (EC: 3.6.1.15) was differentially expressed in the leaves of *C. subternata* that were cultivated under different water stress conditions (Figure 3c). This enzyme is in the plasma membrane and plays a role in breaking down extracellular triphosphate nucleotides [67].

## 4. Materials and Methods

### 4.1. Plant Material and Sample Collection

**Figure 4:**
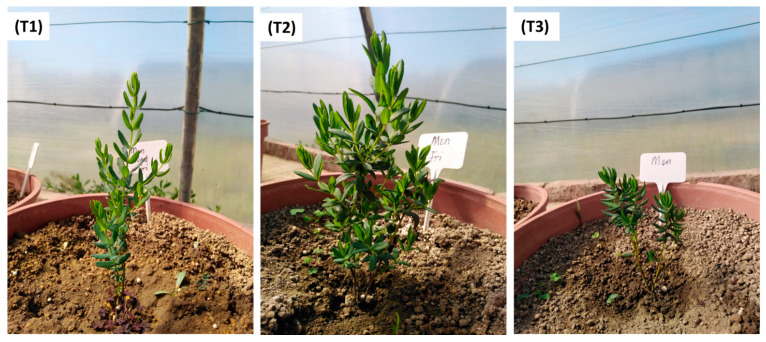
Photos of irrigated and stressed *Cyclopia subnernata*, (**T1**) = Nietvoorbij well-watered plant sample (3 days/week, Control), (**T2**) = Nietvoorbij semi-stressed plant sample (watered 2 days/week), and (**T3**) = Nietvoorbij water-deprived plant sample (watered 1 day/week).

To assess the effect of water deficit stress, a full-scale experiment was set up with potted *C. subternata* plants in a controlled glasshouse at the Agricultural Research Council (ARC), Infruitec, Stellenbosch (−33.925920°, 18.874259°), South Africa. The experiment was conducted for 112 days (from mid-May until early September 2021). All plants were watered uniformly with 300 mL of water for the first 81 days after transplanting (DAT) to ensure uniformity and strong root growth before treatment application. Thereafter, *C. subternata* plants were subjected to three irrigation treatments (from early August to early September 2021) until the study was terminated at 112 DAT. The watering cycles were irrigating thrice (3 days/week), twice (2 days/week), and once (1 day/week), which is described further as T1 = Nietvoorbij well-watered plant sample (Control), T2 = Nietvoorbij semi-stressed plant sample, and T3 = Nietvoorbij water-deprived plant sample (Figure 4). At every irrigation time, 300 mL of water was applied per pot. Each treatment was replicated eight times. The water needed per potted plant (300 mL) was based on extensive in-house preliminary trials.

Under semi-/mild-stressed water conditions, plants showed early vigor or maturity development (Figure 4, T2). Under this water stress condition, the plant undergoes low evapotranspiration to optimize water use efficiency and limit the loss of water due to direct evaporation from the soil surface. The early vigor enables water to be stored and made available later for developmental processes as the soil water becomes gradually depleted (Tuberosa, 2012) [68]. Furthermore, according to Seleiman et al. [69], plants have developed diverse adaptive strategies through evolution that make them more tolerant to the adverse effects of water stress. This survival strategy includes drought stress escape, avoidance, and tolerance approach. Based on the observed morphological changes in the semi-stressed *C. subternata*, escape and tolerance water stress survival strategy responses could be hypothesized. However, further investigation would be needed to validate this hypothesis.

Furthermore, the glasshouse experiment was compared to a commercially managed honeybush farm (Napier farm in Cape Agulhas, South Africa). The commercial farm was managed under good agricultural practice (GAP) with regular irrigation regimes, and the plants were never under any water stress. Samples were collected from the farm in May 2021, and batches were collected based on the year the honeybush was cultivated between 2013 and 2019; T13 = Napier well-watered plants cultivated in 2013, T17 = Napier well-watered plants cultivated in 2017, and T19 = Napier well-watered plants cultivated in 2017. Leaf samples were collected into pre-marked centrifuge tubes and immediately stored in liquid nitrogen. Samples were immediately sent to the Proteomics Research & Services Unit of the University of the Western Cape for protein extraction, 1D- SDS PAGE analysis, HILIC digestion, and other analysis.

### 4.2. Sample Preparation

Frozen samples were ground to a fine powder in liquid nitrogen using a mortar and pestle. Ground samples were stored at −20 °C until used. Ground tissue (0.5 g) containing 0.025 g polyvinylpolypyrrolidone (PVPP) was resuspended in 2 mL of 10% TCA/acetone. Polyvinylpolypyrrolidone (PVPP) has a high capacity to bind to polyphenols, and it is effective for the removal of phenolic impurities from plant tissue extracts [70]. The samples were thoroughly vortexed for 30 s and centrifuged at 16,000× *g* for 3 min at 4 °C. The resultant pellet was further rinsed with 2 mL of cold acetone and centrifuged for 3 min at 16,000× *g* at 4 °C. Acetone rinses were repeated until a white pellet was obtained. The protein pellet was air-dried at room temperature and further used for protein extraction.

### 4.3. Protein Extraction and Pellet Solubilization

Proteins were extracted using the phenol/SDS extraction protocol described previously by Wang et al. [71] with slight modifications. The protein pellet was resuspended in 0.7 mL SDS extraction buffer (30% sucrose, 2% SDS, 100 mM Tris-HCl, pH 8.0, 2.5% 2-mercaptoethanol, 1 mM PMSF) and 0.7 mL phenol (Tris-buffered, pH 8.0; Sigma-Adrich, St. Louis, MO, USA). The mixture was vortexed for 20 min and the phenol phase was separated by centrifugation at 16,000× *g* for 15 min at 4 °C. The phenol phase was backextracted with an equal volume of SDS extraction buffer for 3 min followed by centrifugation at 16,000× *g* for 10 min at 4 °C. At least 5 volumes of cold methanol containing 0.1 M ammonium acetate was added to the phenol phase, and the mixture was stored at −20 °C for 16 h. Precipitated proteins were recovered at 16,000× *g* for 20 min at 4 °C and washed with cold methanol once, followed by two 80% acetone washes. The final pellet was air-dried and dissolved in 100 µL protein solubilization buffer (4 M urea, 2% SDS, 50 mM Tris-HCl, pH 8.0), and the protein concentration was quantified using the Pierce microplate BCA protein assay kit (Thermo Scientific, Rockford, IL, USA) according to the manufacturer’s instructions with bovine serum albumin used as a standard.

### 4.4. Quality Control Using SDS–PAGE Analysis

The purity and quality of the extracted proteins were evaluated using SDS-PAGE analysis. Briefly, proteins (10 µg) were prepared in a 1:3 ratio with 4× Laemmli SDS-PAGE buffer (250 mM Tris-HCl, pH 6.8; 4% SDS; 30% glycerol; 350 mM β-mercaptoethanol; 0.02% bromophenol blue) and boiled at 95 °C for 3 min. The proteins were then resolved according to their molecular weight on 12% polyacrylamide gels under constant 100 V with the aid of the Mini—Protean III^®^ Cell gel casting system (Bio-Rad Laboratories Ltd., Rosebank, Johannesburg, South Africa) until bromophenol blue reached the bottom of the gel. After electrophoresis, proteins were visualized using the Acqua Stain protein gel dye, and the gels were processed using Quantity One software on the Molecular Imager PharosFX Plus System (Bio-Rad Laboratories Ltd., Rosebank, Johannesburg, South Africa).

### 4.5. Protein Pellet Solubilization

All protein pellets were first solubilized in 50 mM Tris containing 2% SDS (Sigma-Adrich, St. Louis, MO, USA) and 4 M urea (Sigma-Adrich, St. Louis, MO, USA) by vortexing for 30 min. Samples were quantified using the Thermo-Fischer BCA kit following the manufacturer’s instructions. Approximately 50 µg of protein was aliquoted for trypsin digestion.

### 4.6. On-Bead Digest

All reagents used are of analytical grade or equivalent. Samples were resuspended in 50 mM ammonium bicarbonate (Sigma-Adrich, St. Louis, MO, USA) before reduction with 10 mM dithiothreitol (DTT) (Sigma) for 30 min at room temperature. This step was followed by alkylation with 30 mM iodoacetamide at room temperature in the dark. After the reduction and alkylation of the protein samples, the samples were diluted with an equal volume of binding buffer (200 mM sodium acetate, 30% acetonitrile, pH 4.5). The protein solution was added to MagResyn HILIC magnetic particles (Resyn Biosciences (Pty), Ltd. Gauteng, South Africa) prepared according to the manufacturer’s instructions and incubated overnight at 4 °C. After binding, the supernatant was removed, and the magnetic particles were washed twice with washing buffer (95% acetonitrile). After washing, the magnetic particles were suspended in 50 mM ammonium bicarbonate containing trypsin (New England Biolabs^®^, Ipswish, UK) to a final ratio of 1:50. After overnight incubation at 37 °C, the peptides were removed from the beads and collected in a fresh tube. The adsorbed peptides were removed by incubating them for 3 min at room temperature in 20 µL 1% trifluoroacetic acid (TFA).

Residual digest reagents were removed using Empore Octadecyl C18 extraction discs (SupelcoTM Analytical, Sigma-Adrich, St. Louis, MO, USA) as the C18 stage tip. The samples were loaded onto the stage tip after methanol (30 µL) equilibrated with 2% acetonitrile: water, 0.05% TFA (30 µL), was used to activate the C18 membrane. The bound sample was washed with 2% acetonitrile: water, 0.1% TFA (30 µL), and thereafter eluted with 50% acetonitrile: water 0.05% TFA (30 µL). The eluate was evaporated to dryness, and the dried peptides were dissolved in 2% acetonitrile: water, 0.1% FA, for LC-MS analysis.

### 4.7. LC–MS/MS Analysis—Dionex Nano-RSLC

The method for LC–MS/MS analysis was adapted from [72]. Thermo Scientific Ultimate 3000 RSLC equipped with a 5 mm x 300 µm C18 trap column (Thermo Scientific, Rockford, IL, USA) and a Charged Surface Hybrid (CSH) 25 cm × 75 µm of a 1.7 µm particle size C18 analytical column (WatersTM, Microsep Pty Ltd., Johannesburg, South Africa) was used for liquid chromatography. The loading solvent system employed was 2% acetonitrile: water, 0.1% FA; Solvent A: 2% acetonitrile: water, 0.1% FA; and Solvent B: 100% acetonitrile: water. The samples were loaded onto the trap column using a loading solvent at a flow rate of 2 µL/min from a temperature-controlled autosampler set at 7 °C. Loading was performed for 5 min before the sample was eluted onto the analytical column. The flow rate was set to 250 nL/min, and the gradient was generated as follows: 5–35% solvent B over 60 min and 35–50% solvent B from 60 to 75 min. Chromatography was performed at 40 °C, and the outflow was delivered to the mass spectrometer through a stainless-steel nano-bore emitter.

#### 4.7.1. Mass Spectrometry

Mass spectrometry was performed using a Thermo Scientific Fusion MS fitted with a Nanospray Flex ionization source. The sample was introduced through a stainless-steel emitter. Data were collected in positive mode with spray voltage set to 1.8 kV and ion transfer capillary set to 280 °C. Spectra were internally calibrated using polysiloxane ions at *m*/*z* = 445.12003 and 371.10024. MS1 scans were performed using the orbitrap detector set at 120,000 resolutions over the scan range 350 to 1650 with an AGC target at 3 × 10^5^ and a maximum injection time of 50 min. Data were acquired in profile mode.

The MS2 acquisitions were performed using monoisotopic precursor selection for ions with charges +2 to +7 with error tolerance set to ±10 ppm. Precursor ions were excluded from fragmentation once for a period of 60 s. Precursor ions were selected for fragmentation in HCD mode using the quadrupole mass analyzer with HCD energy set to 30%. Fragment ions were detected in the orbitrap mass analyzer set to 30,000 resolutions. The AGC target was set to 5 × 10^4^ and the maximum injection time to 80 min, and data were acquired in centroid mode.

#### 4.7.2. MS Data Analysis

The raw files generated via MS were imported into Proteome Discoverer v1.4 (Thermo Scientific, Rockford, IL, USA) and processed using the Sequest and Amanda algorithms. Database interrogation was performed against a concatenated database created using the Uniprot “Fabaceae-reviewed” (accessed in December 2021). Semi-tryptic cleavage with 2 missed cleavages was allowed. Precursor and fragment mass tolerance was set to 10 ppm and 0.02 Da, respectively. The deamidation (NQ), oxidation (M), and acetylation of protein N-terminal were allowed as dynamic modifications and thiomethyl of C as a static modification. Using the Target-Decoy PSM validator node, the peptides were validated. The search results were imported into Scaffold Q+ for further validation (www.proteomesoftware.com, accessed on 14 December 2021).

### 4.8. Gene Ontology and KEGG Analysis Pipeline

The proteins identified were mapped to Universal Protein Resource (UniProt https://www.uniprot.org/id-mapping, accessed 22 August 2022) to assess their function. Functional annotations of differentially abundant proteins (DAPs) were performed using Panther for gene ontology analysis (http://www.pantherdb.org, accessed 17 February 2023). The proteins were classified by Gene Ontology annotation based on three categories: biological process, cellular component and molecular function [73]. Moreover, the DAPs sequences were uploaded into EggNog-Mapper genome-wide functional annotation (http://eggnog-mapper.embl.de, accessed 18 February 2023). Sequences were assigned to various metabolic pathways using the Kyoto Encyclopedia of Genes and Genomes (KEGG) pathway analysis via EggNog-Mapper [74].

### 4.9. Experimental Design and Statistical Analysis

A full factorial experimental layout design was used in this study. The treatments were set up in a randomized block design (RBD) with three different irrigation treatments. All proteomics analyses were conducted using independent replicates in triplicate (*n* = 3). To test for normality, the Shapiro–Wilk test was performed. Data obtained were analyzed using ANOVA at *p* ≤ 0.05, and mean values were tested according to Tukey’s multiple comparison test at *p* ≤ 0.05, with F-values considered significant at *p* ≤ 0.01. Protein quantitation was performed using Fischer’s exact test at (*p* < 0.001) on the paired data with the Benjamini–Hochberg correction applied. Protein identifications were accepted if they could be established at greater than 95% probability, with a protein threshold of 1% false discovery rate, and contained at least two unique identified peptides.

## 5. Conclusions

This study demonstrated that water deficit stress had a critical influence on the biological processes, molecular activities, and cellular compartments of *C. subternata*. Only 11 differentially expressed proteins (DEPs) were found to be unique across all the treated and control *C. subternata*. The DEPs identified were associated with the biological processes linked to the cellular process, metabolic process, and response to a stimulus. Based on the KEGG analysis, five molecular pathways were identified, namely, phenylpropanoid biosynthesis, glyoxylate and dicarboxylate metabolism, purine metabolism, thiamine metabolism, and carbon fixation in a photosynthetic organism. Within these pathways, five enzymes (ribulose biphosphate carboxylase, glutamine synthetase, nucleoside-triphosphate phosphatase, heme-thiolate, and cinnamyl alcohol dehydrogenase) were identified. Overall, the use of proteomic tools helped identified proteins in honeybush plants that are water-stress-relevant. Future investigations to assess the mechanisms responsible for making *C. subternata* water-stress-tolerant compared to other crop species are required.

## Figures and Tables

**Table 1:** List of proteins identified from *C. subternata* plants grown under well-watered and water-stressed conditions.

Protein Name	Cellular Component	Molecular Functions	Biological Processes	MW (kDa)	Tax. ID	FET	* FC (log2)	FC	Expression Change
Alpha-glucan phosphorylase, H isozyme	Cytoplasm	Glycogen phosphorylase activity, pyridoxal phosphate binding, SHG alpha-glucan phosphorylase activity, linear malto-oligosaccharide phosphorylase activity	Carbohydrate metabolism	95.9	3906	0.00070	0.5	1.41	Upregulated in T17 and downregulated in T19
Ribulose bisphosphate carboxylase large chain	Plastid, Chloroplast	Magnesium ion binding, monooxygenase activity, ribulose-bisphosphate carboxylase activity	Photorespiration, reductive pentose-phosphate cycle	50	49830	0.00070	1.1	2.14	Upregulated in T1 and downregulated in T19
Trans-cinnamate 4-monooxygenase	Integral component of membrane	Heme binding, iron ion binding, trans-cinnamate 4-monooxygenase activity,	Lignin metabolic process	58	3847	0.00029	0.0	1.00	Downregulated in T1 and upregulated in T19
Probable UDP-arabinopyranose mutase 1	Extracellular region (Secreted, cell wall, cell junction, plasmodesma, Golgi apparatus)	UDP-arabinopyranose mutase activity	Cell wall organization, cellulose biosynthetic process, plant-type cell wall organization or biogenesis, protein glycosylation	-	3888	0.00081	0.1	1.07	Downregulated in T1 and upregulated in T19
Probable cinnamyl alcohol dehydrogenase	Stem, hypocotyl, root tissue	Cinnamyl-alcohol dehydrogenase activity, sinapyl alcohol dehydrogenase activity, zinc ion binding	Lignin biosynthetic process	-	3879	0.0037	0.09	1.06	Downregulated in T1 and upregulated in T19
Ribulose bisphosphate carboxylase large chain	Plastid, Chloroplast	Magnesium ion binding, monooxygenase activity, ribulose-bisphosphate carboxylase activity	Photorespiration, reductive pentose phosphate cycle	50	49830	0.00074	1.2	2.30	Downregulated in T19 and upregulated in T3
Ribulose bisphosphate carboxylase large chain	Plastid, Chloroplast	Magnesium ion binding, monooxygenase activity, ribulose-bisphosphate carboxylase activity	Photorespiration, reductive pentose phosphate cycle	50	49830	0.00081	1.2	2.30	Downregulated in T19 and upregulated in T3
Ribulose bisphosphate carboxylase large chain	Plastid, Chloroplast	Magnesium ion binding, monooxygenase activity, ribulose-bisphosphate carboxylase activity	Photorespiration, reductive pentose-phosphate cycle	53	49830	0.0015	1.2	2.30	Downregulated in T19 and upregulated in T3
Glutamine synthetase nodule isozyme	Cytoplasm	ATP binding, glutamate-ammonia ligase activity	Glutamine biosynthetic process	39	3918	0.0018	0.7	1.62	Upregulated in T19 and downregulated in T3
Elongation factor 1-alpha	Cytoplasm	GTP binding, translation elongation factor activity, GTPase activity	-	49	3918	0.0037	1.6	3.03	Downregulated in T19 and upregulated in T3
Chlorophyll a-b binding protein, chloroplastic	Chloroplast thylakoid membrane, integral component of membrane, photosystem I, photosystem II	Chlorophyll binding, metal ion binding	Photosynthesis, light harvesting in photosystem I, response to light stimulus	26	3847	0.0041	0.6	1.52	Upregulated in T19 and downregulated in T3

T1 = well-watered Nietvoorbij field plants; T3 = water-stressed Nietvoorbij field plants; T17 = well-watered Napier field plants cultivated in 2017; T19 = well-watered Napier field plants cultivated in 2019 (UniProt https://www.uniprot.org, accessed 22 August 2022). * FC = Fold change, FET = Fisher’s Exact Test, MW = Molecular weight, Tax. ID = Taxonomic identifier.

## Data Availability

The data generated in this work will be made available by the corresponding authors upon request.
